# Detection of *human papillomavirus *DNA and *p53 *codon 72 polymorphism in prostate carcinomas of patients from Argentina

**DOI:** 10.1186/1471-2490-5-15

**Published:** 2005-11-24

**Authors:** Gustavo J Leiros, Silvia R Galliano, Mario E Sember, Tomas Kahn, Elisabeth Schwarz, Kumiko Eiguchi

**Affiliations:** 1 Catedra de Bioquimica e Inmunologia, Facultad de Medicina-Universidad del Salvador, Buenos Aires, Argentina; 2 Servicio de patología, Hospital Israelita, Buenos Aires, Argentina; 3 Deutsches Krebsforschungszentrum, Heidelberg, Germany; 4 Expert Team Life Sciences, Deutsche Bank AG, Frankfurt, Germany

## Abstract

**Background:**

Infections with high-risk human papillomaviruses (HPVs), causatively linked to cervical cancer, might also play a role in the development of prostate cancer. Furthermore, the polymorphism at codon 72 (encoding either arginine or proline) of the *p53 *tumor-suppressor gene is discussed as a possible determinant for cancer risk. The *HPV *E6 oncoprotein induces degradation of the p53 protein. The aim of this study was to analyse prostate carcinomas and hyperplasias of patients from Argentina for the presence of *HPV *DNA and the *p53 *codon 72 polymorphism genotype.

**Methods:**

*HPV *DNA detection and typing were done by consensus L1 and type-specific PCR assays, respectively, and Southern blot hybridizations. Genotyping of *p53 *codon 72 polymorphism was performed both by allele specific primer PCRs and PCR-RFLP (Bsh1236I). Fischer's test with Woolf's approximation was used for statistical analysis.

**Results:**

*HPV *DNA was detected in 17 out of 41 (41.5 %) carcinoma samples, whereas all 30 hyperplasia samples were *HPV*-negative. Differences in *p53 *codon 72 allelic frequencies were not observed, neither between carcinomas and hyperplasias nor between *HPV*-positive and *HPV*-negative carcinomas.

**Conclusion:**

These results indicate that the *p53 *genotype is probably not a risk factor for prostate cancer, and that *HPV *infections could be associated with at least a subset of prostate carcinomas.

## Background

Prostate cancer is one of the most common malignancies in males, but little is known about the molecular events involved in its development [1]. The prostate could constitute a target for infection with *human papillomaviruses *(*HPV*) due to anatomical reasons, particularly by direct access of the viral particles through the urethra. Penile and urethral *HPV *lesions have been described [2], as well as an increased prostate cancer risk associated with sexual behaviour [3]. Several studies have shown the presence of *HPV *DNA in prostate carcinomas and hyperplasias [4,5], whereas others could not detect any [6]. Thus, the possible role of *HPV *in prostate carcinogenesis is still unclear.

The carcinogenic potential of high risk *HPV *types (such as *HPV16 *and *HPV18*) is largely determined by the two oncoproteins E6 and E7. A major function of E6 is to bind and to target the tumor-suppressor protein p53 for proteosomal degradation [7], whereas E7 inactivates the retinoblastoma protein pRb [8]. There exists a polymorphic sequence in the *p53 *gene at codon position 72 encoding either arginine (Arg) or proline (Pro) [9]. It has been reported that the p53 protein with Arg (p53-Arg72) is more susceptible to E6-mediated degradation than the proline form (p53-Pro72) and that the Arg allele is over-represented in cervical cancer patients [10]. The conclusion that the *p53*-Arg72 allele confers a higher risk for cervical cancer development than the *p53*-Pro72 allele has either been supported by subsequent studies [11] or not [12]. On the other hand, it has been shown that Pro homozygosity is associated with a reduced risk of prostate cancer [13], and therefore this allele could have some protective effect.

In this study we have analyzed prostate neoplasia samples of patients from Buenos Aires, Argentina, in order to evaluate the prevalence of *HPV *DNA and the distribution of *p53 *codon 72 alleles. We have tried to minimize the possibility of urethral HPV contaminations by using microdissection for further sample processing before DNA extraction.

## Methods

### Studied Population

89 caucasian men older than 60 years were studied from whom 41 had prostate adenocarcinoma diagnosis and the 48 remaining had hyperplasia diagnosis. All patients were from Buenos Aires city, Argentina.

Helsinki recommendations for tissue sampling were observed. In addition, we had scientific committee approvals from institutions involved in the present report.

### Clinical samples

Samples of histopathologically confirmed adenocarcinomas and benign hyperplasias were obtained by biopsy (transrectal prostatic puncture method). Three to five specimens (puncture biopsy) were obtained from each patient, fixed in formaldehyde-phosphate buffer, embedded in paraffin, and slides from these pieces were stained with hematoxylin-eosin for histopathological analysis. Blood samples from each patient were also obtained by venous puncture and collected in tubes with EDTA.

### Dissection of neoplastic tissue

Specimens with hyperplasias or infiltrated by adenocarcinoma cells were first selected. In a second selection process the areas corresponding to carcinoma or hyperplasia were microdissected in order to obtain samples with the highest percentage of neoplastic cells. Slides and hematoxylin-eosin staining from these new fragments were performed to confirm the success of the procedure. These steps were repeated as many times as necessary to obtain microscopic images showing more than 90% of neoplastic cells.

### DNA extraction

Genomic DNA from deparaffinized tumor samples and peripheral blood cells was obtained by proteinase K digestion, followed by phenol-chloroform extraction and ethanol precipitation. To assess the quality of the isolated DNA for PCR, a 268 bp long segment of the β-globin gene was amplified by PCR using the primers GH20 and PC04. Only DNA samples showing specific amplification with this set of primers were used for *HPV*- and/or *p53*-specific PCR assays. Due to the small sizes of many biopsies and the low amounts of DNA extracted, it was not possible to perform both *HPV *and *p53 *PCR experiments with all samples.

### Detection and typing of *HPV *DNA by PCR and hybridization

DNA samples of all 41 prostate carcinomas and of 30 prostate hyperplasias were available for *HPV *analysis. DNA of the 18 remaining hyperplasia samples was completely used up for the *p53 *PCR analysis. As described in Hoffmann et al. [14], DNA was first analyzed for the presence of *HPV *sequences by multiplex PCR with type-specific (TS) primers for *HPV *types 6, 11, 16 and 18 (Table 1). TS-PCR-negative samples and samples for which only very small amounts of DNA were available were subjected to PCR with the consensus L1 primers MY09 and MY11, able to recognize a wide range of mucosotropic *HPV *types (Table 1). PCR reactions were performed in a Peltier Thermal Cycler 2000 DNA Engine (MJ Research Inc., Watertown, Massachusetts, USA). The reaction conditions for TS-PCR were as follows: initial denaturation at 94°C for 5 minutes, 39 cycles with denaturation at 94°C for 1 minute, annealing at 54°C for 2 minutes and elongation at 72°C for 2 minutes. In the last cycle, the elongation step was extended to 10 minutes. The reaction conditions for PCR amplification with the consensus primers were identical, with the exception that annealing was performed at 55°C for 1 minute. In each PCR reaction we took precautions to an extreme in order to avoid contaminations with PCR products. For this purpose we manipulated both reagents and products in completely separated rooms, and used disposable materials and different sets of instruments. Furthermore, a negative control (water instead of DNA) was included in each set of PCR reactions.

**Table 1:** Oligonucleotides used as primers and radiolabelled probes for *HPV *type-specific and consensus PCR.

**Oligonucleotide**	**Nucleotide sequence**	**Localization in *HPV *genome**
TS-*HPV6*-1	+5'-TAGTGGGCCTATGGCTCGTC-3'	*E5*: 4671–4690
TS-*HPV6*-2	-5'-TCCATTAGCCTCCACGGGTG-3'	*E5*:4931–4950
TS-*HPV6 *probe	+5'-CATTAACGCAGGGGCGCCTGAAATTGTGCC-3'	*E5*: 4761–4790
TS-*HPV11*-1	+5'-GGAATACATGCGCCATGTGG-3'	*L1*: 6841–6860
TS-*HPV11*-2	-5'-CGAGCAGACGTCCGTCCTCG-3'	*L1*: 7181–7200
TS-*HPV11 *probe	+5'-CGCCTCCACCAAATGGTACACTGGAGGATA-3'	*L1*: 6977–7006
TS-*HPV16*-1	+5'-TGCTAGTGCTTATGCAGCAA-3'	*L1*: 6028–6047
TS-*HPV16*-2	-5'-ATTTACTGCAACATTGGTAC-3'	*L1*: 6160–6179
TS-*HPV16 *probe	+5'-CAAACCACCTATAGGGGAACACTGGGGCA-3'	*L1*: 6117–6146
TS-*HPV18*-1	+5'-AAGGATGCTGCACCGGCTGA-3'	*L1*: 6903–6922
TS-*HPV18*-2	-5'-CACGCACACGCTTGGCAGGT-3'	*L1*: 7100–7119
TS-*HPV18 *probe	+5'-TGGTTCAGGCTGGATTGCGTCGCAAGCCCA-3'	*L1*: 7021–7050
MY11	+5'-GCMCAGGGWCATAAYAATGG-3' (W = A+T, Y = C+T; M = A+C)	*L1*: 6582–6601
MY09	-5'-CGTCCMARRGGAWACTGATC-3' (W = A+T; R = A+G; M = A+C)	*L1*: 7033–7014

**Consensus probe:**		

MY18	+5'-CTGTTGTTGATACTACACGCAGTAC-3'	*L1*
MY46	+5'-CTGTGGTAGATACCACWCGCAGTAC-3'	*L1*
MY57	+5'-CTGTGGTAGATACCACACGTAGTA-3'	*L1*
WD147	+5'-CTGTAGTGGACACTACCCGCAGTAC-3'	*L1*

For each experiment, 150 ng of DNA from the patient sample was used together with 50 pmoles of each primer, 0.01 μmoles of each dNTP, 1.5 mM of MgCl_2 _and 2 units of Taq DNA polymerase in reaction buffer (GIBCO BRL-Life Technologies Inc Gaithersburg, MD, USA). *HPV*-positive and negative control reactions were done in parallel in all experiments. In the *HPV *type-specific PCR assays, the *HPV*-positive controls included genomic DNA of SiHa (*HPV*16-positive cell line), and C4-I (*HPV*18-positive cell line), as well as cloned DNA of *HPV*6 and *HPV*11. In the consensus L1 PCR, genomic SiHa DNA was used as *HPV*-positive control. In both types of PCR assays, DNA from the *HPV*-negative cell line HaCaT was used as *HPV*-negative control. PCR products were subjected to electrophoresis on 2% agarose minigels, visualized by ethidium bromide staining and blotted on Type B positive nylon membranes (Fluka Chemie AG, Buchs, Switzerland). Southern hybridization was performed with the radiolabelled oligonucleotide probes shown in Table 1. Filter hybridization, washing, and exposure as well as 5'-end labelling of oligonucleotide probes were done as described [14].

### PCR assays for *p53 *polymorphism at codon 72

PCR was performed as described in Storey et al. [10] with tumor and peripheral blood cell DNA. For the *p53 *PCR, DNA of 39 prostate carcinomas (the DNA of 2 samples was completely used up for the *HPV*-specific PCR assays) and 48 prostate hyperplasias was available. Two sets of primer pairs (Table 2) were used for detection of *p53*-Pro72 and *p53*-Arg72 sequences, respectively. The different variants could be discriminated by the different sizes of PCR products (Table 2). The PCR conditions were as follows for the *p53*-Pro72 allele: denaturation at 94°C for 5 minutes, then 35 cycles with denaturation at 94°C for 1 minute, annealing at 56°C for 1 minutes and elongation at 72°C for 1 minute. In the last cycle, the elongation step was extended to 10 minutes. For the *p53*-Arg72 allele, the PCR conditions were identical with the exception that annealing was performed at 62°C for 1 minute. The PCR products were separated in 3 % agarose gels. Possible assay outcomes were: 1) if a PCR product (136 bp) was obtained only with the arginine-specific primers, the patient was considered arginine homozygous, 2) if only a proline-specific primer product (178 bp) was obtained, the patient was considered proline homozygous, 3) if the sample showed amplification with both two primer sets, the patient was considered heterozygous (Arg/Pro).

**Table 2:** PCR primers used for the analysis of *p53 *polymorphism at codon 72.

**Allele**	**Primer**	**Primer sequence**	**PCR product size**
*p53*-Pro72	*p53 *Pro +	5'GCCAGAGGCTGCTCCCCC3'	178 bp
	*p53*-	5'CGTGCAAGTCACAGACTT3'	

*p53*-Arg72	*p53*+	5'TCCCCCTTGCCGTCCCAA3'	136 bp
	*p53 *Arg-	5'CTGGTGCAGGGGCCACGC3'	

A second assay for *p53 *polymorphism status was performed using a RFLP (restriction fragment length polymorphism) site for the enzyme Bsh1236I (5'-CGCG-3'), present in the Arg allele (CGC-G), but not in the Pro allele (CCC-G) [9]. PCR reactions were performed with the *p53*^+ ^and *p53*^- ^external primers (table 2), using *p53*-Pro72 allele PCR conditions, amplifying a product of 279 bp length. The PCR product was then digested with 10 U of Bsh1236I (Fermentas GmbH, St. Leon-Rot, Germany) during 90 minutes at 37°C. Digestion products were run in 3% agarose gels. In case of the Arg allele, cleavage products of 160 bp and 119 bp were obtained.

### Statistical analysis

Statistical analysis was performed using exact Fisher's test with Woolf's approximation. Statistical analysis was performed with Statistica 5.0 software program (Stat Soft Inc Tulsa, OK, USA).

## Results

### Presence of *HPV *DNA in prostate tissues

**Figure 1:**
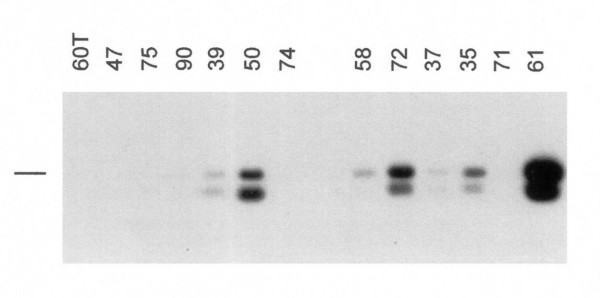
***HPV *DNA detection in prostate carcinomas**. PCR reactions using primers MY09 and MY11 were performed. After gel electrophoresis and transfer, the filter was hybridized with consensus probes. Positive (SiHa) and negative (HaCaT, water) controls were included in the experiment, but are not shown in the figure. The numbers above the lanes indicate the designation of the carcinoma samples. The bar on the left side indicates the position of the MY09/11 PCR product of approximately 450 bp length. Two hybridization signals are visible in the *HPV*-positive samples. The upper band corresponds to the MY09/11 PCR product. The additional lower band which was also seen in other experiments using these batches of the MY09/11 primers, appeared exclusively as a companion of the *HPV*-specific 450 bp product. We did not try to clarify the nature of this extra-band.

To assess the presence of *HPV *DNA in prostate lesions, DNA of histopathologically confirmed samples of 41 prostate carcinomas and 30 prostate hyperplasias was analyzed. The tumor sections were obtained by microdissection in order to minimize contamination with stromal tissue. Using multiplex PCR and Southern blot for *HPV *types 6, 11, 16 and 18, five *HPV16*-positive and 2 *HPV11*-positive prostate carcinomas were detected, whereas all benign prostate hyperplasias were negative (Table 3). The *HPV*-negative samples were subjected subsequently to PCR with the MY09/MY11 consensus primers and Southern blot with consensus probes (Figure 1). This assay detected 10 additional *HPV*-positive carcinoma samples, whereas all benign hyperplasias remained negative (Table 3). Unfortunately, no *HPV *typing could be performed with the MY09/MY11-positive samples mainly due to the lack of additional DNA material. Statistical analysis indicated a significant association (Fischer's exact test with Woolf's approximation, p < 0.0001) between *HPV *DNA presence and prostate carcinomas.

**Table 3:** *HPV *DNA in prostate carcinomas and hyperplasias.

**Samples**	***HPV+***				***HPV-***
		
	***HPV 16***	***HPV 11***	***HPV *Consensus^a^**	***HPV *+ total**	
**Prostate carcinomas (n = 41)**	5^b^	2	10	**17**^c^	**24**
**Prostate hyperplasias (n = 30)**	0	0	0	**0**	**30**

^a ^samples were negative in the TS-PCR, but *HPV*-positive in the MY09/MY11 PCR.. ^b ^p = 0.068, Fischer's Exact Test with Woolf's approximation; ^c ^p < 0.0001, Fischer's Exact Test with Woolf's approximation.

### *p53 *polymorphism at codon 72

**Figure 2:**
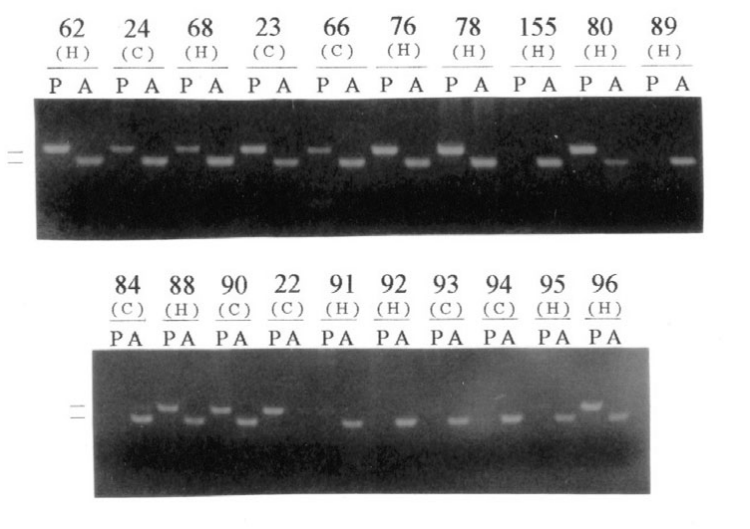
**Analysis of *p53 *codon 72 polymorphism in prostate carcinomas and hyperplasias by allele-specific PCR**. The PCR products were run on a 3 % agarose gel. Numbers above the lanes indicate the designation of the tumor samples. (H) and (C) denote prostate hyperplasia or carcinoma, respectively. A or P indicate the use of Arg or Pro specific primer sets, respectively. The bars on the left side indicate the positions of the PCR products for Arg allele (136 bp) and Pro allele (178 bp).

In parallel to the *HPV *studies, we have analyzed the *p53 *polymorphism at codon 72 (Arg, Pro or Arg/Pro) in the leukocyte and tumor DNA from 39 patients with prostate carcinomas and 48 patients with prostate hyperplasias by allele-specific PCR and PCR-RFLP analysis. The results of the allele-specific PCR are shown in Figure 2. The two methods gave consistent results for each DNA, and no differences were detected between the tumor and normal DNA of each patient. The data are summarized in Table 4. Among the 39 prostate cancer patients, 20 Arg homozygotes, 2 Pro homozygotes, and 17 Arg/Pro heterozygotes were identified. From the hyperplasia patients, 23 were Arg homozygotes, 2 Pro homozygotes, and the 23 remaining heterozygotes. For the statistical analysis, the *p53 *Pro allele-carrying patients (Pro homozygotes and Arg/Pro heterozygotes) were grouped together and compared with the Arg homozygotes, in order to evaluate the latter genotype as risk factor. In the frequency of *p53 *Arg homozygosity no significant differences (Fischer's exact test with Woolf's approximation, p = 0.831) could be detected between carcinoma and hyperplasia patients.

**Table 4:** Genotypes and frequencies of codon 72 *p53 *polymorphism variants in prostate carcinomas and hyperplasias.

***p53 *codon 72 allele**	**Prostate Carcinomas**	**Prostate Hyperplasias**
Arg/Arg	20 (0.51)	23 (0.48)
**Pro/Pro**	2 (0.05)	2 (0.04)
**Pro/Arg**	17 (0.44)	23 (0.48)
**Total**	39	48

The data are a summary of all experiments because identical results were obtained for each DNA with the two methods used for *p53 *allele typing (allele specific PCR, and PCR-RFLP), and no differences were observed between the two DNA sample sources (peripherical blood leucocytes or neoplastic tissue) from the individual patients.

Next, we compared the *p53 *codon 72 allelic frequencies between patients with *HPV*-positive and *HPV*-negative carcinomas in order to evaluate whether an association between *p53 *Arg homozygosity and *HPV*-positivity might exist. From 17 patients with *HPV*-positive carcinomas, 9 were Arg homozygotes, 2 were Pro homozygotes and 6 were Arg/Pro heterozygotes. From the 22 patients with *HPV*-negative carcinomas, 11 were Arg homozygotes, none were Pro homozygotes and 11 were heterozygotes (Table 5). For the Fisher's test the samples were grouped in the same way as described above. No significant differences (Fischer's exact test with Woolf's approximation, p = 1.00) in the frequency of *p53 *Arg homozygosity could be observed between *HPV*-positive and *HPV*-negative prostate carcinomas.

**Table 5:** Genotypes and frequencies of codon 72 *p53 *polymorphism variants in *HPV*-positive and *HPV*-negative prostate carcinomas

***p53 *codon 72 allele**	**Prostate Carcinomas**	
	
	***HPV *(+)**	***HPV *(-)**
**Arg/Arg**	9 (0.53)	11 (0.50)
**Pro/Pro**	2 (0.12)	0 (0)
**Pro/Arg**	6 (0.35)	11 (0.50)
**Total**	**17**	**22**

## Discussion

In the analysed sample collection we have detected a great difference in *HPV *positivity between prostate carcinomas (17 out of 41 = 41,5 %) and hyperplasias (0 out of 30 = 0 %) From the 7 carcinomas with identified *HPV *type, 5 samples contained the high-risk *HPV*16 and 2 samples the low-risk *HPV*11. The presence of *HPV*16 DNA supports the assumption that high-risk *HPV *infections are associated with at least a subset of prostate cancers. The presence of *HPV*11 DNA points to the possibility that HPV can infect the prostate, but these infections have probably no influence on the carcinogenic process.

After more than 10 years of *HPV *DNA analysis in benign and malignant prostate samples, the causal involvement of HPV in prostate carcinogenesis is still a matter of controversial debate. The discrepant results and methodological problems of the earlier analyses have already been discussed in Cuzick [5] and Strickler et al [6]. It has been speculated that the discrepancies could be due to *HPV *contamination from nearby tissues during the sampling procedure since *HPV *DNA has been detected in urethral [15,16] and anal [17,18] tissues. Based on these data some authors recommended radical prostatectomy as tissue source, as well as an exhaustive microdissection of the neoplastic sample. For the present study radical prostatectomy samples could not be obtained. However, we have performed a microdissection approach to exclude contaminating anal tissue as well as to minimize stromal content from the samples. On the other hand, if a *HPV *contamination from anal epithelium is a common event during biopsy taking and sample manipulation, it would be expected that both carcinomas and hyperplasias show some degree of *HPV *detection. However, we could not detect any *HPV *DNA in the hyperplasias. In some recent studies, *HPV *DNA was either detected in prostate cancer samples [19] or not [20,21]. Possible explanations for the divergent frequencies of *HPV*-positivity in prostate cancer samples may be found in populational, geographical, environmental and genetic heterogeneities, beyond methodological detection problems.

In cervical cancer, several studies of the *p53 *codon 72 polymorphism have been performed after the initial report claiming a higher cancer risk associated with the Arg allele [10]. Some of them refute the original finding [22-24] whereas others support it [25,26]. In our analysis of the *p53 *polymorphism at codon 72, we could not find an indication that the Arg allele confers a higher risk for prostate cancer, including those tissues positive for *HPV*. The use of two different typing methods and polymorphism determination, in both blood and tumor samples, avoid misinterpretations due to methodological typing problems and LOH in cancer samples. A recent study came to the conclusion that the Pro/Pro genotype is associated with a reduced risk of prostate cancer [13]. We could not evaluate this hypothesis due to the extremely low populational frequency of the rare Pro/Pro genotype.

It will remain important issues for future studies of prostate carcinogenesis to assess the presence, expression and potential role of *HPV *and to further understand the contribution of *p53 *mutations and polymorphisms.

## Conclusion

In the present work, *HPV *DNA was detected in 17 out of 41 (41,5 %) prostate cancer samples, whereas all 30 tested benign hyperplasias were *HPV*-negative. The results allow the conclusion that HPV infections might be associated with prostate carcinoma development, at least in a subset of cases. In addition, the allelic frequencies of the *p53 *codon 72 polymorphism (Arg, Pro or Arg/Pro) were determined in the patients with benign and malignant tumors in order to evaluate the possibility of increased cancer susceptibility associated with the Arg allele. However, no statistically significant differences in the Arg and Pro (Pro plus Arg/Pro) allelic frequencies could be detected, neither by comparing patients with carcinomas and hyperplasias nor between *HPV*-positive and *HPV*-negative carcinomas.

## Competing interests

(1) The authors declare that they have no competing interests.

(2) Financial resources expended during the assays were supported by PICT0528 Grant from National Agency for Scientific and Technology Promotion (Argentina), ARG 99/029 mobility Grant from the International Bureau of the Federal Ministry of Education, Science, Research and Technology (BMBF, Germany) and the Secretary for Technology, Science and Productive Innovation (SETCIP, Argentina), and Alberto J Roemmers Foundation Grant.

## Authors' contributions

GJL carried out the molecular genetic studies, performed the statistical analysis and drafted the manuscript. SRG carried out the pathological diagnosis and microdissected the biopsy samples. MES carried out the prostatic biopsies and contributed with clinical urologic knowledge. TK participated in the design of the study. ES participated in the design of the study, gave continuous technical support and helped to draft the manuscript. KE planned the study, and participated in its design and coordination. All authors read and approved the final manuscript.

## Pre-publication history

The pre-publication history for this paper can be accessed here:


